# The Impact of Demographic Characteristics on Parenting Stress among Parents of Children with Disabilities: A Cross-Sectional Study

**DOI:** 10.3390/children11020239

**Published:** 2024-02-13

**Authors:** Maxi Scheibner, Cora Scheibner, Frauke Hornemann, Maria Arélin, Yvonne Doris Hennig, Henriette Kiep, Ulrike Wurst, Andreas Merkenschlager, Janina Gburek-Augustat

**Affiliations:** Division of Neuropediatrics, Hospital for Children and Adolescents, University Hospital Leipzig, 04103 Leipzig, Germany; cora.scheibner@medizin.uni-leipzig.de (C.S.); andreas.merkenschlager@medizin.uni-leipzig.de (A.M.); janina.gburek-augustat@medizin.uni-leipzig.de (J.G.-A.)

**Keywords:** parenting stress, disabled children, demographic characteristics, parenting stress index, impact on family scale

## Abstract

Even though it is already known that parents of children with developmental delays or disabilities experience higher parenting stress than families of typically developing children, the contributing factors need to be analyzed in more detail. The aim of this cross-sectional study was to examine the influence of demographic characteristics on parenting stress from caring for a disabled child and to identify possible protective or additional stressful social factors. A total of 611 mothers and fathers of children with developmental delays, chronic diseases, or disabilities completed two questionnaires during their medical appointments at the Children’s Development Center (CDC) of Leipzig University Hospital between June 2020 and February 2021. These consisted of the German versions of the Parenting Stress Index (PSI) and the Impact on Family Scale (IOFS). To determine differences between the various groups, we used parametric and non-parametric tests. Mothers and single parents are significantly more strained than fathers and non-single parents. Parents with vocational training, those who graduated with a higher-level diploma, and those within employment report a higher financial burden. While unemployed and full-time workers experience the lowest stress, parents who work part-time or exclusively take care of their child show higher levels of stress. Looking at the age of the child, parents of children of young primary school age are the most stressed, and those of infants are the least stressed. These findings suggest that mothers and single parents especially should receive more support, and parents need to be provided with more attention during their child’s entry into school. Possible limitations and the influence of the COVID-19 pandemic are discussed.

## 1. Introduction

About 10 percent of children in Germany live with limitations due to developmental delays, chronic diseases, or disabilities. Every year, approximately 250,000 of them are treated in Children’s Developmental Centers (CDC), where they are accompanied by their parents and receive medical and psychological diagnostics, counseling, and therapy [1].

Children’s disorders can cause serious stress for parents and the whole family. Various studies have shown that parents of children with developmental delays, disabilities, or other diseases are more stressed in different areas of life than parents of healthy and typically developing children [2,3,4,5]. A study in the UK revealed that 72% of these parents suffer from poor mental health with anxiety or depression, 49% take medication or see a counselor, and 65% feel isolated [6]. They have to invest considerably more time in the care and nurturing of their child, while worry is an integral part of their everyday lives [3,7]. However, the greatest stress is exhibited by parents of children with behavioral problems [8,9,10], causing fear of stigmatization and avoidance of public situations, as well as illnesses that require comprehensive and ongoing care for their children [4]. Many families have to change or even give up their careers in order to ensure adequate care for their child [7].

When comparing mothers and fathers, mothers usually report higher stress levels and psychosocial stress as well as greater impairment in everyday life than fathers [11]. Moreover, mothers’ stress levels increase over time, while fathers’ stress levels tend to remain constant [12]. For instance, mothers often have to give up their jobs and report poorer overall health and quality of life than fathers [3,4,13]. Mothers are usually the main caregiver, carry the burden of their child’s disability, and spend most of their time with their child and attend all their child’s appointments [3]. At the same time, fathers and mothers utilize different coping strategies to deal with stressful life situations [4].

Looking at the impact of social factors on parenting stress, there are many contradictory findings. Some studies indicate that sociodemographic factors, such as educational status, do not correlate with parenting stress [14], and a well-functioning family is more important than socioeconomic factors [15]. Other studies have identified differences in parenting stress between social conditions. According to Innocenti et al. [16], mothers with disabled children are more likely to be unemployed, which means a greater strain on the family than in families in which both parents are employed [17]. A higher level of education was associated with better mental health, and being with employment was correlated with greater psychological well-being [18]. At the same time, lower income and education led to increased stress [19] and could even cause delayed care access [15]. There are often financial problems due to health costs, which puts a strain on these families.

The parent partnership can be strengthened by disability and thus protect against higher levels of stress [6], but it can also be weakened, thereby further increasing the level of stress on the family [20]. Also, age, both of the parents and of the child, can have an influence on stress. Thus, it has been reported that older parents had fewer negative effects and feelings than younger parents, indicating that the impact of the child’s disability attenuates with increasing age [18]. Furthermore, some studies have found that parents of young children, especially infants, are less stressed, and that the level of stress increases with the age of the children [16], while other studies reported the highest stress in infancy [21].

The purpose of our CDC in Leipzig, Germany, is to offer parents and children holistic care and support in various problem areas. Based on this, we had the premise that families would experience significant relief through their connection to a multidisciplinary team at the CDC and their stress values would differ significantly from those in the literature, for example, from developing countries or countries without care coordination services [22]. However, an initial evaluation of our data has shown that more than half of our treated parents experience stress within the clinically relevant range and that this is not sufficiently perceived by the attending pediatricians [23]. That is why it is important to better understand all the influencing variables in order to offer these parents the best possible support. Until now, we have found that parents’ stress correlates with greater impairment of the child, but this mainly occurs in combination with behavioral disorders. To determine other vulnerable groups that should be paid more attention to, the aim of this study was to examine the influence of demographic characteristics on parenting stress from caring for a disabled child and to identify possible protective or additional stressful social factors. As studies in other countries are usually associated with different healthcare systems, and therefore there is often unequally distributed access to care services, it is interesting for us to find out what differences exist in our German CDC with its statutory healthcare system. Therefore, we included the factors of age and gender of parents, partner situation, education, vocational training, employment, age of children, and number of siblings as variables for parental and child characteristics. Furthermore, for many years, it has been known that mothers experience significantly more stress from caring for their disabled child than fathers. As a result, there have been increasing demands to distribute family responsibilities more equally between mothers and fathers in order to relieve the burden on mothers. In addition, the location of our CDC in eastern Germany should result in a more balanced employment ratio between mothers and fathers, as mothers in eastern Germany in general work more often, sooner after their parental leave and are more likely to work full-time than mothers in western Germany [24]. We wanted to assess whether there had already been a change due to these conditions. To address these issues, we defined three research questions:

1. Do mothers of impaired children still experience more parental stress than fathers?
2. Are there sociodemographic factors that are associated with increased parental stress?
3. Are there sociodemographic factors that protect against increased parental stress?

## 2. Materials and Methods

### 2.1. Participants

This cross-sectional study includes 611 participants represented by mothers and fathers who have been surveyed. The children of these parents suffer from different disabilities, which show a variety of diagnoses, including developmental delay, consequences of prematurity, epilepsy, Down syndrome, and many other syndromes, as well as neuromuscular diseases.

### 2.2. Procedure

Parents came with their children to their appointment during the consultation hours of the CDC at Leipzig University Hospital between June 2020 and February 2021. Some of the families were attending their first appointment, while others had been coming to regular consultations for years. In order to become a patient at the CDC, children are referred by their treating outpatient pediatricians. Reasons for this include an existing impairment, deviant development of the child, or risk of developing a disability. Before a child receives an appointment, one of the pediatricians first checks whether there is a clear indication for the referral. At the CDC, they are offered detailed diagnostics, advice, and treatment by an interdisciplinary team consisting of pediatricians, physiotherapists, occupational therapists, speech therapists, psychologists, and social workers. In this way, parents and children can improve their competencies in defined target areas, helping them to cope with their disability and contributing to the best possible integration and participation. Most of the children came with a known diagnosis. Children who had not yet received a diagnosis were assigned a diagnosis based on the ICD-10-GM criteria. At this CDC, the participating parents filled out the questionnaires before the consultation, with only one of the parents completing it. First, they were introduced to the questionnaires, and their consent was obtained. Then, they answered them on their own. For families to whom German was a foreign language, an interpreter occasionally came to help if they brought one with them.

In this study, a convenience sampling strategy was employed. We aimed to include all parents who came to their consultation between June 2020 and February 2021 and agreed to their participation with written consent. As the families regularly attend their appointments every quarter or no later than every six months, we chose the above period for our survey to reach the majority of patients. Our exclusion criteria included non-German speakers who did not have an interpreter available and children who could not be diagnosed with a disability or developmental delay.

Of 710 parents who came to the CDC during the period, 611 answered the questionnaires. The participation rate was 86%. Non-participants were either not interested (34.5%) or there was a language barrier (65.5%).

Each questionnaire was assigned a multidigit number in order to anonymize it. This was communicated to the parents prior to their participation to protect data privacy and avoid socially desirable responses.

### 2.3. Measures

Detailed descriptions of questionnaires are given in Appendix A.

#### 2.3.1. German Version of Parenting Stress Index (PSI)

The Eltern-Belastungs-Inventar questionnaire [25] is the German version of Abidin’s (1983) PSI published in 2010. It is used to record parent–child interaction and the resulting stress on parents. First, demographic data of the respondent and the child are requested, for example age, school-leaving qualification, occupation, and number of siblings. The actual main part of the questionnaire consists of 48 items, with 4 items for each of the 12 subscales, assessed on a 5-point Likert scale. It is mainly divided into two categories: the child domain and the parent domain. Here, the child domain comprises 5 subscales and captures characteristics of the child around the themes of hyperactivity, acceptability, demandingness, adaptability, and mood. In the parenting domain, the 7 subscales include social isolation, personal restriction, parental competence, health, attachment, depression, and partner relationship, which show the parents’ impairment. All subscales are aggregated to a total score. Higher values in the scales represent higher levels of stress. For better comparability of the scales, all scores are normalized using T values. Thus, a value above 60 then indicates a high strain and a value above 70 a very high strain. The questionnaire was tested in a study with German mothers of disabled or chronically ill children and adolescents and showed a Cronbach’s alpha between 0.91 and 0.95 and very good retest reliability results.

#### 2.3.2. German Version of Impact on Family Scale (IOFS)

The Familien-Belastungs questionnaire [17] is an instrument used to assess the impact of children’s chronic disease on family stress developed in 2001. It was created on the basis of the English version “Impact on Family Scale”. The questionnaire consists of 33 items assigned to the following 5 subscales: daily social stress (15 items), financial burden (4 items), burden on siblings (6 items), personal strain (5 items), and coping (3 items). Finally, an overall score is calculated from all categories except concern of siblings. To answer the items, there is a 4-point Likert scale from 1, meaning “does not apply at all”, to 4, denoting “applies completely”. Higher scores mean greater stress on families. The questionnaire was tested on 273 German families with chronically ill children within one study and showed reliability values between 0.7 and 0.89, as well as very good validity.

### 2.4. Data Analysis

Data were analyzed using IBM^®^ SPSS Statistics 27th version. Descriptive statistics were collected first. We used analysis of variance (ANOVA) and Student’s *t*-test for normally distributed data as in the German version of IOFS. The Kruskal–Wallis H-test and Mann–Whitney U-test were utilized for non-normally distributed data used in the German version of PSI. To test correlations of categorical variables, we included cross-tabulations and the chi-square test. Therefore, we categorized the data into various groups based on sociodemographic characteristics (e.g., mothers and fathers) and compared them for significant differences. To compare only two groups, we used the Mann–Whitney U-test for the German version of PSI data and the *t*-test for the German version of IOFS data. If more than two groups were tested for differences, we used the Kruskal–Wallis H-test for the German version of PSI and ANOVA for the German version of IOFS. In cases where there were significant differences between more than two groups, we checked these pairwise using a post hoc analysis. We performed the Bonferroni test for the ANOVA and the Dunn–Bonferroni test for the Kruskal–Wallis H-test. The significance level was defined as *p* < 0.05. Missing data or incompletely finished questionnaires were excluded.

### 2.5. Ethical Considerations

The study was conducted in accordance with the Declaration of Helsinki and approved by the Ethics Committee of the University Leipzig with protocol number 027/20-ek. The participating parents were informed of how their data would be used and had the right to withdraw their participation at any time.

## 3. Results

### 3.1. Descriptive Statistics

Participant demographics are shown in Table 1. There were more mothers (*n* = 484; 79.2%) than fathers (*n* = 127; 20.8%), and the parents’ age ranged from 17 to 58 years (M = 37.72; SD = 6.73). The majority were between 30 and 39 years old (*n* = 325; 53.2%). Three quarters of the parents were two-parent families, and one quarter were single parents. A total of 88.7% had a partner and 11.3% lived alone with their child or children. School education in our study is higher than average [26], with the majority having completed the highest-track schooling (*n* = 261; 42.7%), and the minority had no degree (*n* = 16; 2.6%) or a compulsory school diploma (*n* = 88; 14.4%). In total, 40.3% (*n* = 246) finished middle-track school. Almost 90% graduated from vocational training or university. When we conducted interviews, two-thirds of the respondents were in employment in addition to their parenthood. Precisely 2.8% were in training or studying, 37.3% were working part-time, and 24.7% were working full-time. The remaining parents did not have a job, with 13.1% unemployed, 18.7% exclusively caring for their child or being on parental leave, and 3.4% having some other reason such as early retirement. Separated by gender, markedly more mothers worked part-time (43.2%), while only 15% of fathers did so. The latter, in contrast, mostly worked full-time (55.9%), and mothers (21.9%) cared for their child more often than fathers (6.3%).

Regarding child characteristics, there were more boys (*n* = 361; 59.1%) than girls (*n* = 250; 40.9%). They ranged in age from 1 month to 17 years (M = 6.8; SD = 4.296) and had on average 1.26 siblings.

### 3.2. Statistical Analysis

More than half of the parents (55%) reported stress within the clinically relevant range. Here, parents are more often stressed in the child domain than in the parent domain. For the German version of IOFS, the highest values were found in the ‘personal strain’ category. More detailed information on general stress in our study has been described in a further publication [23].

Table 2 shows differences in parenting stress by parental and children’s characteristics.

Mothers reported more stress in all main scores, especially significantly higher scores in the German version of IOFS total score and subcategories of the parenting domain ‘social isolation’, ‘health’, and ‘personal limitation’. Only in the category ‘parental attachment’ did fathers suffer significantly more stress.

In the German version of IOFS total score, single parents show significantly higher values. They also reached higher scores in the other main categories, but they did not reach a significant level.

Parents of different school degrees differ significantly in the child domain and German version of IOFS total scores. The post hoc analysis, however, could not find any significant differences between the groups for the child domain total score. In the subcategories of ‘adaptability’ and ‘mood’, significant disparities were detected between parents with a middle-track school and highest-track school diploma (adaptability, *p* = 0.013) and between parents with a compulsory school and highest-track school diploma (mood, *p* = 0.033), with parents with highest-track school diploma reporting the lower values in both cases. There were significant differences in the post hoc analysis for the German version of IOFS as well, but here the results are opposite. In the total score (*p* < 0.001) and in the subcategories ‘daily social stress’ (*p* = 0.04) and especially ‘financial burden’ (*p* < 0.001), parents with a compulsory school diploma differ significantly from parents with a middle-track school diploma and those with a highest-track school diploma. Both the values for total stress and those for financial burden (see Table 3) increase with a higher level of education.

Regarding completed vocational training or university, there are significant differences in the parenting domain and German version of IOFS total scores. Those who had successfully completed them report higher stress levels (except in the ‘burden on siblings’ category). Again, paradoxically, we see a higher financial burden among parents with vocational training or a university degree.

Furthermore, parents were asked about their current employment situation. Here, the various groups differ significantly in the German version of IOFS total scores and subcategories as well as the German version of PSI parent domain subcategories of ‘social isolation’, ‘health’, ‘personal restriction’, and ‘partner relationship’. The post hoc analysis revealed significant differences for the German version of IOFS total score and subcategories between unemployed and part-time working parents (*p* = 0.007) and parents caring for their child or on parental leave (*p* = 0.01), as well as between part-time and full-time working parents (*p* < 0.001) and between full-time working parents and parents caring for their child (*p* < 0.001). In the ‘daily social stress’ subcategory, there was an additional significant contrast between unemployed and caring parents (*p* < 0.001), as well as caring parents and parents working part-time (*p* < 0.001). Parents working part-time and those caring for their child each showed higher values, while parents working full-time and the unemployed showed lower values. For the German version of PSI subcategories, the post hoc analysis only identified a significant difference in the ‘personal restriction’ category between parents working full-time (lower) and parents caring for their child (higher, *p* = 0.026). Interestingly, significant disparities were again found for the ‘financial burden’ category between unemployed (lower) and part-time working parents (higher, *p* < 0.001), caring parents (higher, *p* = 0.012), part-time working parents (higher), and full-time working parents (lower, *p* < 0.001), and between part-time working parents (higher) and caring parents (lower, *p* = 0.005).

We also categorized the age of the children into different age groups. These groups can be found in Table 1. Significant differences were found in the child domain total score and the ‘hyperactivity’ subcategory. The post hoc analysis then revealed a significant contrast in the child domain total score between parents of infants and parents of children between 4 and 7 years of age (*p* = 0.038), with infants having lower scores. In the ‘hyperactivity’ subcategory, the comparison of parents of children between 1 and 3 years and between 4 and 7 years was significant (*p* = 0.006), meaning that parents of children between 4 and 7 years again had the higher values. This distribution can also be seen in other categories, but without reaching a significant level.

There are no significant differences in stress between the various numbers of siblings the affected child has. However, there is a slight tendency for the stress on parents to decrease as the number of siblings increases.

Other factors, such as partnership and age of parents, do not show significant differences.

### 3.3. Coherence Test

To test for associations between individual factors, we used cross-tabulations and the chi-square test. Here, a connection between the gender of the parents and the characteristic of single parenthood is evident. Mothers are significantly more often single parents (25%) than fathers (10.2%) (*p* < 0.001). In the same way, there is a significant link between vocational training and employment. Parents who have not completed vocational training are also more likely to not be working (58.8%) than parents who have completed vocational training (31.6%) (*p* < 0.001).

## 4. Discussion

This quantitative cross-sectional study explored the parental stress of parents of children with developmental delays or disabilities in relation to their sociodemographic characteristics. Parents with higher educational attainment are overrepresented in the sample, as in most studies based on written questionnaires. In addition, considerably more parents in our study worked part-time (37.3%) than is the case in the general population of Germany (13.5%). However, the probable reason for this is that there are more women than men in our study, and the proportion of part-time workers is significantly higher among women (47.9%) than among men (11.2%) [27].

Regarding our first research question, we looked at the parental stress experienced by mothers compared to that of fathers. The results showed that mothers reported higher stress levels than fathers, a finding consistent with previous studies [12,13,28]. This could be due to the fact that mothers still spend more time with their child and are also more often responsible for caring and looking after their child in general. According to Gerstein et al. [12], mothers more often self-identify with the role of educator and thus as the primary caregiver for the child, whereas fathers see themselves as the breadwinner and provider. Even today, childcare is often the task of mothers and causes their stress [3]. In past years, there have been growing efforts to even out this disparity between fathers and mothers. In Germany, for example, the percentage of fathers on parental leave has increased since 2015 [29]. Nevertheless, and surprisingly, these changes do not seem to be sufficient to relieve mothers and thus reduce their stress. Remarkably, despite social efforts to relieve mothers, there seem to have been no changes in recent years. That is why it is still important to find solutions to distribute parental responsibilities equally between mothers and fathers. Furthermore, it would be very interesting to look closer at the reasons for the lack of change in this area and whether it can be found in other countries. Families could benefit if fathers would be more integrated into everyday clinical and family life without seeing mothers automatically as the primary caregiver and thus distributing the burden equally on both [30]. Greater involvement of the father not only leads to an improvement in the mother’s well-being but can also have a positive influence on the child’s development [31].

However, the coherence test also revealed that more mothers were single parents, which could thus cause more stress.

With our second research question, we aimed to identify the sociodemographic factors that are associated with increased parental stress. In general, the single parent factor has proven to be stressful. Especially in family life and coping with everyday life, single parents show higher stress levels and are thereby more dependent on social support from relatives and friends. Previous studies have shown that social support from the parents’ environment and professional support are inversely correlated with their stress [32], which makes it even more important to provide them with help. Our data further indicated that parents who cared for their child or worked part-time were significantly more stressed than parents who worked full-time or were unemployed. Children who need to be nursed by their parents are probably suffering from a severe illness or disability and consequently are very affected. Full-scale care of the child requires a lot of time from the parents and leaves them little freedom themselves, which creates a great level of stress [7]. This finding is consistent with other studies, showing that social isolation and physical overload are the main stressors for these parents [33]. Moreover, parents working part-time reported more stress than those working full-time. This stress could result from the conflict that these parents have in terms of working part-time in order to have enough time to take care of their child and thereby having to endure financial losses. Thus, they reported the highest financial worries.

Parents of 4–7-year-old children, especially 7-year-olds, are the most stressed. While some studies suggest that parenting stress decreases as children get older [34], other studies show that a lower quality of life is found at older ages [35]. At the age between 4 and 7 years, preschool and school begin for many children, confronting them with new challenges. Child and parents identify new deficits, and the child presumably needs more support and promotion for learning. According to Coffey [3], the first critical time for parents is around diagnosis, and the second is at important milestones for the child, such as school entry.

In summary, several variables were found to increase parental stress. These include being a single parent, caring for one’s child full-time, working part-time, and parenting a child at school entry age.

Our third research question focused on protective sociodemographic factors that lead to lower parental stress levels. Parent partnership, though, failed to show a significant impact on stress in contrast to other studies [12]. It did not prove to be a protective factor. But this might be because the questions that make up the partnership subcategory in the German version of PSI do not ask about partnership as a relieving factor. They only inquire about additional stress caused by the neglect of the partnership due to the child’s disability. To look closer at the influence of a partnership, then, other questions would have to be chosen. That those working full-time experience less stress is consistent with other studies [18,36]. So, working full-time every day seems to lead to lower stress levels. This suggests that taking a break from childcare tasks and spending time on other activities may protect parents from acute stress. On the other hand, perhaps children of full-time working parents have less severe disabilities, which is what allows them to have this time to work. Using the diagnostic categories from our other publication [23], more children with severe disabilities can be found among part-time workers than among full-time workers (see Appendix B).

Previous studies have often showed an association between lower education and income and higher stress levels [19], as well as higher education levels leading to less stress [37]. Our results also show this distribution in the child domain of the German version of PSI. However, this distribution is reversed in the IOFS. Furthermore, it emerged that parents of higher school graduates, those with vocational training, or who are employed report a higher financial burden compared to those who are unemployed, a finding inconsistent with other studies [6]. Even if higher income (supposed to be more prevalent in higher social positions) seems to be protective for higher quality of life [38], especially in the group of families of these social positions, worries about financial threats are more prevalent. But as questions were asked about subjective financial burdens and not clear numbers, such an assessment may be due to social status. People are more likely to socialize with others of the same status [39], and comparison is primarily made within the social group. So, an academic may have higher expectations and invest more money in improvements for the child. In addition, families of a higher social position presumably are confronted with the prejudice that disability means economic hardship [40]. That can cause a subjective higher financial burden. Also, another study has found that cash subsidy programs are used primarily by lower-income families [41].

Parents of infants show the lowest levels of stress. Some other studies have shown similar results [16], including an increased financial burden as the age of the child increases [17]. The probable reason for this is that parents do not yet realize the deficits at this age, and their child may appear unimpaired. On the other hand, some questions in the questionnaires we used are not well adapted to infants, so parents may be unable to answer them correctly, generating lower scores. Overall, partnership showed no effect on parental stress, while working full-time and caring for an infant appeared to be associated with lower stress levels.

### 4.1. Clinical Implications

Even parents of children with developmental delays or disabilities who are integrated into a CDC and receive multidisciplinary care experience high levels of stress in various areas of life and should be properly supported. Especially single mothers, parents who work part-time or care for their child, and parents whose children are about to start school need particular attention. They could benefit from a range of supports and services such as emotional support, counseling, and the provision of information to improve their quality of life [7]. At the same time, children could benefit from early support services or therapies such as occupational therapy. However, even today, there are still major gaps regarding the provision of care for affected families. They often complain about a lack of offers, limited accessibility in rural areas, low financial support, a high level of bureaucratic effort, and repeated rejections of their applications [42]. These barriers have to be reduced in the future to offer these families the best possible assistance.

Furthermore, both pediatricians and nurse practitioners are encouraged to be aware of the signs of increased stress when treating these higher-risk groups and to ask them explicitly about their areas of conflict. Only by working with these families can the tensions in parent–child interaction be recognized and explored in detail.

Since working full-time seems to be protective, parents who want to work again should be aided in doing so, so that they can find a balance between this and alleviate worries for their children. For this, more childcare offers would be needed.

Various interventions that have been developed in recent years have shown that it is possible to reduce parents’ stress and increase their psychological well-being [43]. For example, a “Parent Child Relationally Informed—Early Intervention” program was able to reduce the significantly elevated stress levels of parents of children with developmental delays recorded in the PSI below the clinically critical level [44]. Such programs may also be applied to families with children with other disabilities to help them cope with stress and increase their competencies.

Since not all affected areas of parents’ lives can be covered in the general procedures of the consultation, such questionnaires are very well qualified, for example, to create a stress profile of the parents and thereby show them much more suitable support options. In this way, questionnaires could be integrated into daily diagnostics for better care. For instance, these could be handed over to parents by the attending nurse practitioners before their consultation and evaluated by pediatricians or nurses later.

### 4.2. Limitations

When using questionnaires, there is often a central tendency bias in order to achieve social desirability that may skew the results. In addition, the two questionnaires used were selected by researchers. Other questionnaires with alternate wording may lead to different results. As this is a hospital-based study, the selective choice of study participants may have created a bias. Some non-German speakers were not included, and the data only represent a specific region and hospital in Germany. This makes it difficult to generalize our findings to all parents of children with disabilities. But since our CDC represents a wide catchment area in the tenth largest city in Germany, the findings might be representative of other parts of the Western world, and thus also for North America and Europe.

What must be considered is that the results of our study may have been influenced by the COVID-19 situation and lockdowns during the interviews. Parents had to invest much time in homeschooling and preventive care, and children also suffered from these restrictions and showed behavioral problems, sleep problems, and mood swings [45]. This may have increased the stress levels we measured. Another factor that could have influenced parents’ stress levels is their health status. Unfortunately, we did not collect these data using questionnaires. Moreover, the COVID-19 situation may have led to poorer physical and mental well-being of the parents and thus affected the data. In Germany, there was an increase in depressive symptoms, anxiety, and a deterioration in subjective mental health from the end of 2020 [46]. Nevertheless, the highest values were found in 2022, meaning they were obtained after completing our study.

The variables considered in our study did not include all factors that may influence parental stress. That is why we cannot clearly say whether our variables really have an influence on the results or if they are dependent on other variables. It would therefore be interesting to extend this research to further variables. Since the questionnaires we used did not examine the impact of parents’ partnership thoroughly enough, further investigation could help to understand the contradictory results of previous studies. Other relevant variables would be the exact income of parents to better assess their financial burden, support from the social environment, which support services are already used, if there is an inclusion of the children in kindergarten or school, and specific disorders of the children.

In conclusion, this study indicates that mothers, single parents, parents who work part-time or care for their child, and parents of 7-year-old children show the highest levels of clinically relevant stress. Further, parents in presumably higher socioeconomic positions report the highest subjective financial worries. Stress may be assessed by standardized measures, such as questionnaires, in order to provide parents with the best possible support. Reducing parents’ stress can not only lead to improvements in their mental health but also strengthen the relationship with their disabled child, contributing to his or her good development. Since there have been no substantial changes in recent years, further measures and research are needed.

## Figures and Tables

**Table 1:** Participants demographics.

Parent Characteristics (*n* = 611)	Group	
Gender, *n* (%)	Mother	484 (79.2%)
	Father	127 (20.8%)
Age, years (mean, sd)		37.7 (6.7)
Single parents, *n* (%)	No	477 (78.1%)
	Yes	134 (21.9%)
Relationship, *n* (%)	No	69 (11.3%)
	Yes	542 (88.7%)
School education, *n* (%)	Not graduated	16 (2.6%)
	Compulsory schooling only (9 years)	88 (14.4%)
	Middle-track school (10 years)	246 (40.3%)
	Highest-track school (12 years)	261 (42.7%)
Vocational training/University, *n* (%)	No	80 (13.1%)
	Yes	531 (86.9%)
Employment, *n* (%)	Unemployed	80 (13.1%)
	In education/training	17 (2.8%)
	Part-time	228 (37.3%)
	Full-time	151 (24.7%)
	Child care/parental leave	114 (18.7%)
	Other	21 (3.4%)
Employed, *n* (%)	No	215 (35.2%)
	Yes	396 (64.8%)
Child characteristics (*n* = 611)		
Gender, *n* (%)	Male	361 (59.1%)
	Female	250 (40.9%)
Age, years (mean, sd)		6.8 (4.3)
Age groups, *n* (%)	1–12 months	17 (2.8%)
	1–3 years	131 (21.4%)
	4–7 years	216 (35.4%)
	8–12 years	170 (27.8%)
	13–17 years	77 (12.6%)
Siblings, number (mean, sd)		1.26 (1.2)
Siblings, *n* (%)	0	169 (27.7%)
	1	246 (40.3%)
	2	118 (19.3%)
	3	46 (7.5%)
	>3	32 (5.2%)

SD, standard deviation.

**Table 2:** Differences in parenting stress by parental and children’s characteristics.

Parent and Child Characteristics	Child Domain (Median) ^a^	Parent Domain (Median) ^a^	German Version of PSI Total Score (Median) ^a^	German Version of IOFS Total Score (Mean) ^b^
Gender				*p* < 0.001
Mother	65	57	62	**1.96**
Father	65	53	58	**1.77**
Single parents				*p* = 0.018
No	65	55	61	**1.90**
Yes	66	57.5	63	**2.01**
Relationship				
No	64	58	62	2.00
Yes	65	56	61	1.91
School education	*p* = 0.024			*p* < 0.001
Not graduated	**71**	53	62	**1.87**
Compulsory schooling only (9 years)	**65**	53	58	**1.70**
Middle-track school (10 years)	**66.5**	57	63	**1.95**
Highest-track school (12 years)	**62**	56	61	**1.97**
Vocational training/University		*p* = 0.018		*p* = 0.036
No	62	**52**	57.5	**1.82**
Yes	65	**57.5**	62	**1.94**
Employment				*p* < 0.001
Unemployed	66	54	59	**1.79**
In education/training	66	58	62	**1.88**
Part-time	64	57	63	**2.20**
Full-time	65	55	60	**1.73**
Child care/parental leave	66	57,5	62	**2.40**
Other	71.5	62	70	**2.60**
Child age groups	*p* = 0.004			
1–12 months	**56**	48	50	1.89
1–3 years	**61**	57	61	1.98
4–7 years	**67**	57	63	1.93
8–12 years	**66**	54	59	1.87
13–17 years	**64**	54	61	1.97
Siblings				
0	65	56	61	1.95
1	66	57	62	1.91
2	65	57	63	1.95
3	59	53	57	1.93
>3	65	51	55	1.69

^a^ Mann–Whitney U-test and Kruskal–Wallis H-test. ^b^ *t*-test and ANOVA.

**Table 3:** Differences in financial burden by parental characteristics.

Parent Characteristics	Financial Burden (Mean)	*p* ^a^
Vocational training/University		<0.001
No	1.69	
Yes	2.05	
School education		<0.001
Not graduated	1.72	
Compulsory schooling only (9 years)	1.52	
Middle-track school (10 years)	2.05	
Highest-track school (12 years)	2.13	
Employment		<0.001
Unemployed	1.58	
In education/training	1.83	
Part-time	2.31	
Full-time	1.82	
Child care/parental leave	1.97	
Other	1.90	

^a^ *t*-test and ANOVA.

## Data Availability

The data presented in this study are available on request from the corresponding author. The data are not publicly available due to privacy reasons.

## Appendix A

### Appendix A.1. German Version of Parenting Stress Index (PSI)

The Eltern-Belastungs-Inventar (EBI) by H. Tröster [25] is the German version of the Parenting Stress Index (PSI), which was established by R. R. Abidin in 1983 [47] First applied in 2010, it serves as a screening method for high parenting stress, providing initial indications of the need for support. Based on Abidin’s parenting stress model, it is hypothesized that parenting stress is primarily caused by the child’s challenging and difficult behavior and parental restraints that complicate successful coping. It includes several questions about the sociodemographic characteristics of the respondents and 48 items about parental stress. First, gender, age, nationality, education, and current occupational status of the parent are recorded, as well as the existence of a partnership and the number of children. Then, 48 statements from 12 subscales (4 per scale) are presented for parents to answer using a 5-point Likert scale, where “1” means strongly disagree and “5” strongly agree. These 12 subscales can be divided into two main domains:

(1) Child domain: This domain gathers information on child-related sources of stress and contains the five following subsections: child’s distractibility/ hyperactivity, acceptability, demandingness, adaptability, and mood.
(2) Parent domain: This mainly evaluates seven subsets of parental constraints: parental attachment, social isolation, competence, depression, parental health, role restriction, and partner relationship.

All raw values were transferred into stanine or T values using a numerical norm table. High values represent high parenting stress. In the 12 subscales, stanines rank between 1 and 9. At a value ≥ 7 (≥77th percentile), the scale developers assume parenting stress in a clinically relevant range. The three total scales of child domain, parent domain, and total score include T values from <30 to >70. Values ≥ 60 (≥84th percentile) indicate stress in a clinically relevant range, whereas values ≥ 70 (≥98th percentile) represent very high stress levels.

Internal consistency according to Cronbach’s alpha α is 0.95 of the EBI total score, 0.91 of the child domain, and 0.93 of the parent domain. The internal consistency of the subscales is lower (0.61 to 0.83) due to the smaller number of items. Retest reliability after one year is 0.87 for the total score and 0.85 and 0.87 for the child and parent domains. The questionnaire was standardized by means of two surveys of 538 mothers with typically developing children between 1 and 5 years of age in a German kindergarten and childminder study. Based on the stress scores from these surveys, the cut-off values were determined. It was validated by 12 independent German studies conducted between 1998 and 2005, which primarily focused on mothers of children with disabilities between 0 and 21 years of age.

### Appendix A.2. German Version of Impact on Family Scale (IOFS)

The Familien-Belastungs (FaBel) questionnaire [17] represents the German version of the Impact on Family Scale (IOFS) and was developed in 2001. It consists of 33 items based on a 4-point Likert scale, ranging from “1”, strongly disagree, to “4”, strongly agree. It is used to assess parenting stress in families of children with chronic diseases or disabilities and can be divided into five different stress categories: daily social stress (15 items), personal strain (5 items), concern of siblings (6 items), financial impact (4 items), and coping problems (3 items). In addition, a total score can be calculated that reflects the total family stress. This is composed of four of five categories, excluding concern of siblings, to ensure comparability with families with single children. The values of the subscales and the total score ranged from 1.0 to 4.0. A higher score in any domain means a higher level of stress.

To test reliability and validity, a cross-sectional study was conducted with 273 families of children with chronic diseases or disabilities between 0 and 18 years of age. All five subscales achieved Cronbach’s alpha α values greater than or equal to 0.7 for internal consistency. Total score has a reliability of α = 0.89, and the α of subscales was between 0.7 and 0.89. Scale fit is an indicator for the correct assignment of individual items to subscales. It amounts to very good at 85% to 100% for all scales.

## Appendix B

**Table A1:** Percentage distribution of diagnostic categories among the employment situations.

			Diagnostic Categories					
Employment	1	2	3	4	5	6	7	8
Unemployed	11.3%	12.5%	5%	8.8%	20%	22.5%	17.5%	2.5%
In education/training	17.6%	11.8%	11.8%	17.6%	11.8%	23.5%	5.9%	0%
Part-time	16.7%	7.9%	12.7%	7.5%	**18.4%**	**16.2%**	**18.9%**	1.8%
Full-time	**20.5%**	9.3%	10.6%	13.9%	**15.2%**	**13.9%**	**14.6%**	2%
Child care	12.3%	4.4%	12.3%	0.9%	18.4%	21.1%	26.3%	4.4%
Other	23.8%	9.5%	9.5%	4.8%	23.8%	14.3%	9.5%	4.8%

Table A1 Percentage distribution of diagnostic categories among the employment situations. 1. Developmental disorder, 2. mental disability, 3. physical disability, 4. behavioral disorder, 5. mental or physical disability and behavioral disorder, 6. mental and physical disability and behavioral disorder, 7. mental and physical disability without behavioral disorder, 8. other. Table A1 shows the percentage distribution of the different diagnostic categories among the groups of employment statuses.
